# Disseminated hepatic epithelioid hemangioendothelioma with multiorgan metastases achieving long-term stability with Pazopanib: a case report

**DOI:** 10.3389/fonc.2026.1834539

**Published:** 2026-06-03

**Authors:** Alaa Naiem Zaro, Salar Nael Sajadieh, Ansam Khaled Doudin, Salem K. Qupp, Laith Elayan, Ola Abu Laban, Marwan Qabaja, Amaal Alamleh

**Affiliations:** 1 Faculty of Medicine, Al-Quds University, Jerusalem, Palestine; 2 Department of Oncology, Augusta Victoria Hospital, Jerusalem, Palestine

**Keywords:** case report, hepatic epithelioid hemangioendothelioma, immunohistochemistry, metastasis, Pazopanib

## Abstract

**Background/Objectives:**

Epithelioid Hemangioendothelioma (EHE) is a rare malignant vascular neoplasm with an unpredictable clinical behavior and often delayed diagnosis. The malignancy arises from many anatomical sites, with the liver being the most common primary site. This report portrays a young patient who presented atypically with a multifocal hepatic epithelioid hemangioendothelioma (HEHE) and highlights the challenges faced during the diagnosis and management of such rare malignancies.

**Methods:**

A 28-year-old male patient from Palestine presented with progressive headache associated with constitutional symptoms. Diagnostic imaging revealed disseminated disease throughout the body. CT-guided liver biopsy confirmed neoplastic cells arranged in small nests and individually, some of which showed intracytoplasmic vacuoles within a hyalinized stroma. Immunohistochemistry was positive for CD31, CD34 and FLI-1, supporting the endothelial origin. Metastatic HEHE was established; a biological treatment plan with Pazopanib (Votrient) was initiated, and the patient was followed for 4 years.

**Results:**

During the follow-up period, serial imaging revealed no significant regression or progression of the lesions, indicating stable disease with the treatment used.

**Conclusion:**

The case emphasizes the clinical intricacy of HEHE, given its rarity, the lack of standardized treatment guidelines, and the tumor’s unpredictable course. Specifically, this case illustrates these challenges within the Palestinian healthcare system.

## 1 Introduction

Epithelioid Hemangioendothelioma (EHE) is a rare, low-to-intermediate-grade vascular tumor that arises from endothelial cells (1). According to the World Health Organization (WHO), it is a vascular sarcoma, with an estimated prevalence of less than 1 in 1 million people, affecting females more than males, with a wide range of onset. It was first described by Weiss and Enzinger in 1982 as a vascular tumor that is often misdiagnosed as metastasis from other tumors (2). EHE usually behaves as a low-grade malignancy, although it can originate in virtually any anatomical site, with the lung, liver, and bone accounting for over 65% of reported cases (3, 4). The tumor’s etiology is unknown, but some associations have emerged, including liver trauma, oral contraceptives, vinyl chloride, asbestos, alcohol, viral hepatitis, and contrast agents such as Thorotrast (5). In addition, a causal relationship has emerged between it and chronic Bartonella (6).

EHE is usually asymptomatic in nearly 35% of patients, while the other (~65%) present with metastatic disease at the time of diagnosis. It has a multifocal growth, especially in the liver and lungs (7). When present, symptoms are usually related to the site, with pain and mass effect. In cases of Hepatic Epithelioid Hemangioendothelioma (HEHE), manifestations include right upper quadrant (RUQ) pain, hepatomegaly and weight loss (8). Involvement of major hepatic veins may result in Budd-Chiari syndrome.

Due to its rarity, EHE can be confirmed only by histopathology and immunohistochemical markers (such as CD31, CD34, and vimentin). A high degree of uncertainty can arise in management, and limited therapeutic options are available due to tumor heterogeneity and differences in tumor behavior among patients (9). Of these management lines, radical surgery can be considered especially for HEHE or other conventional therapies (e.g., chemotherapy). It is important to note that radical surgery is not always preferable due to intra- and extra-hepatic extension of the tumor metastasis (2). Liver transplantation (LT) is an effective treatment for HEHE but carries a risk of post-transplant recurrence (10).

Although several case reports have been published in multiple countries, there is a shortage of articles in Palestine. To address this gap, the article presents a case of HEHE with early-onset multiple metastatic lesions, detailing the diagnostic approach and the management plan implemented at Augusta Victoria Hospital in Jerusalem. This article may represent one of the first reported cases of EHE in the country.

The main objective is to consider EHE as one of the differential diagnoses of vascular tumors in Palestine, and to identify which therapeutic options can help reduce the mortality and morbidity from this tumor.

## 2 Case presentation

A 28-year-old male patient from Qalqilya presented with a new-onset headache in May 2021. It was diffuse, persistent, and progressively intensified over the following three months. During that period, the pain was accompanied by constitutional “B-symptoms, “ including night sweats and an unintentional 6-kg weight loss.

His past medical and surgical histories were unremarkable. He had an 8-pack-year smoking history and a documented milk allergy. Family history was significant for malignancy; his father died of lung cancer, one of his paternal uncles was diagnosed with hepatocellular carcinoma (HCC), while another uncle was diagnosed with colonic adenocarcinoma.

Diagnostic imaging was initiated in July 2021 at an outpatient clinic due to the worsening headache and constitutional symptoms. A brain CT was performed first, which provided the initial evidence of multifocal extra-axial disease with significant calvarial involvement and a large space-occupying lesion (SOL) in the left fronto-parietal region. To further characterize these findings, a brain MRI was performed on July 16, 2021. This confirmed a 7.0 × 5.0 × 6.5 cm mass demonstrating intense enhancement, relative central sparing, and prominent vascular signal voids. The mass had breached and eroded the overlying calvarium, extending into the scalp soft tissues and producing a 1-cm midline shift. Subsequent systemic staging with a whole-body contrast-enhanced CT on July 18, 2021, revealed disseminated disease.

Following the identification of widespread malignancy, the patient was referred to Augusta Victoria Hospital, a tertiary oncology center for definitive management. Upon admission in August 2021, he appeared clinically well and hemodynamically stable. A detailed history was obtained as part of the diagnostic workup. A comprehensive examination at our facility was entirely normal, with no focal deficits or psychomotor delay identified (Glasgow Coma Scale 15/15). Abdominal examination was unremarkable, with no palpable organomegaly or tenderness. Cardiac auscultation revealed a “regularly irregular” rhythm, though the patient was asymptomatic.

Given the disseminated nature of the disease, a whole-body CT repeat was performed at the hospital, revealing the following findings:

♦ Abdomen CT: Multiple variable-sized and shaped hepatic lesions, some of which show calcification foci; the largest is seen in the right liver lobe at the end of the tapered right main portal branch (lollipop sign), measuring about 3.7 x 4.6 cm in diameter (**Figure 1A**).
♦ Brain CT scan: a left parietal parafalcine lesion with ring enhancement measuring about 1.1 x 1.2 cm (**Figure 1B**).
♦ Chest CT scan: There are multiple bilateral, variable-sized, and shaped hilar masses and lung nodules; the largest is at the right hilum, measuring approximately 2.8 × 3.2 cm. There is also an enlarged left axillary lymph node measuring 1.7 cm, with a few other prominent lymph nodes (**Figures 1C, D**).
♦ Bone window CT: Multiple bony expansile lesions with internal soft-tissue components are seen at the right iliac bone, the right side of the sacrum, the posterior aspect of the right second rib, and the C7 vertebral body, causing mild canal stenosis at this level (**Figures 2A–C**). In the skull, a few lytic lesions were seen in the left iliac bone, as well as at the vertebral body spinous process of L3, and at the right humeral head. There is a large lytic expansile bony lesion seen in the left temporo-parieto-frontal bone measuring about 5 × 6.2 × 7 cm (**Figure 2D**). Another lytic expansile, smaller lesion with an internal soft-tissue component was seen at the posterior aspect of the right parietal bone, measuring approximately 0.8 × 1.7 cm.

**Figure 1:**
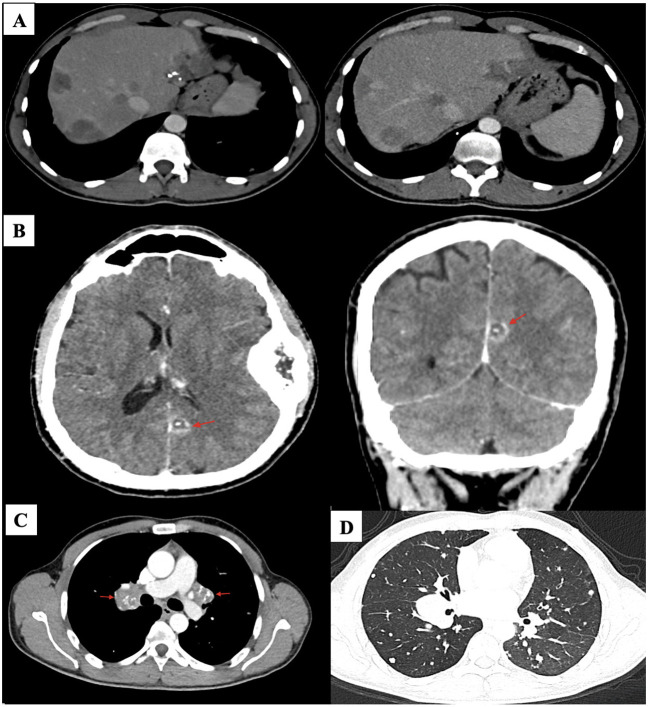
Radiological Findings. **(A)** Axial contrast-enhanced CT images revealed multiple confluent hepatic lesions with predominantly marginal enhancement and central hypoattenuation, some of which show calcification foci. **(B)** Axial and coronal CT scan with IV contrast showing left parietal parafalcine lesion with ring enhancement measuring about 1.1 × 1.2 cm (red arrow). **(C)** Axial contrast-enhanced CT scan demonstrates bilateral hilar masses (red arrows). **(D)** Axial Chest CT (lung window) shows multiple bilateral variable-sized lung nodules.

**Figure 2:**
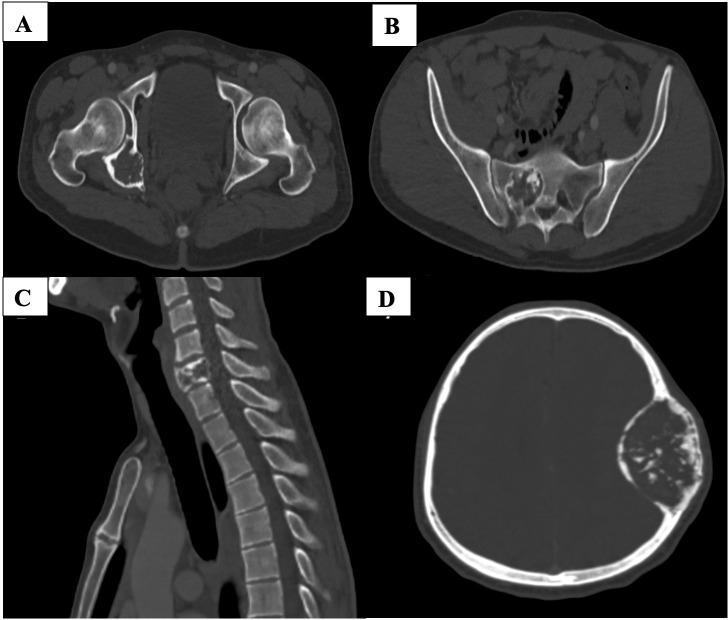
CT scan images (bone window) demonstrate multiple expansile bony lesions with internal soft tissue component and internal foci of calcifications showing marginal sclerosis, e.g.: **(A)** in the right ischium, **(B)** right sacral bone, **(C)** in C7 vertebral body, **(D)** left frontotemporoparietal skull lesion causing severe compression upon the left lateral ventricle and midline shifting to the right side.

Following that, a CT-guided liver biopsy was done, and Histopathologic examination with Hematoxylin and Eosin (H&E) staining revealed a neoplastic proliferation of epithelioid cells arranged individually and in small, cohesive nests. The cells were embedded in a characteristic, dense, hyalinized stroma (**Figure 3A**). A key diagnostic feature was the presence of intracytoplasmic vacuoles within the neoplastic cells, representing primitive vascular lumen formation. With mitotic activity 1 per 10 High Power Field (HPF), and Ki-67 is less than 5%. Immunohistochemical (IHC) analysis confirmed the tumor’s endothelial lineage. The neoplastic cells demonstrated diffuse and robust positivity for the following vascular markers: CD34 (**Figure 3B**), CD31 (**Figure 3C**), and friend leukemia virus integration 1 (FLI-1) (**Figure 3D**).

**Figure 3:**
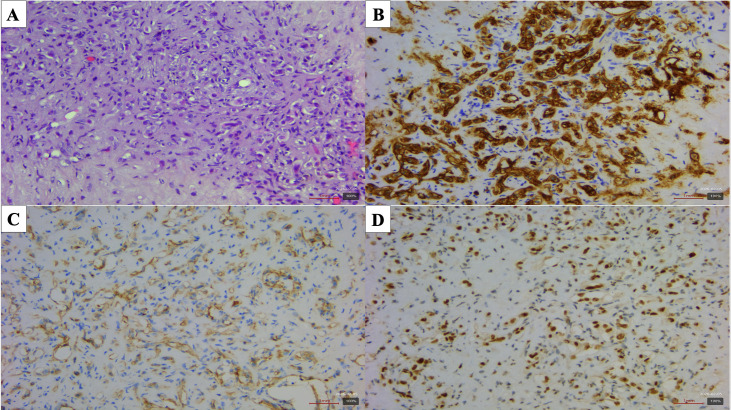
Histopathological findings. **(A)** H&E staining shows neoplastic cells arranged in small nests and individually, some with intracytoplasmic vacuoles, within a hyalinized stroma. **(B)** The neoplastic cells are positive for CD34. **(C)** The neoplastic cells are positive for CD31. **(D)** The neoplastic cells are positive for FLI-1.

Following the histopathological findings (morphological features and IHC profile), the diagnosis of HEHE with widespread visceral and osseous metastases was confirmed. According to that, the patient was initiated on systemic therapy on August 23, 2021, with Pazopanib (Votrient), an oral tyrosine kinase inhibitor (TKI), 800 mg once daily. Given the extensive multi-organ metastatic burden, the treatment strategy focused on long-term systemic control.

The patient was followed up over the subsequent years, initially with serial CT scans. Then, in July 2024, a PET/CT after an IV injection of 8.04 mCi of F18-FDG was performed and considered a baseline. At baseline (July 2024), PET/CT demonstrated FDG-avid lesions in the right hilum, liver, and bone. The largest right hilar lesion measured approximately 4.1 cm with an SUVmax of 6.9. The largest hepatic lesion (segment IVb) measured 2.4 cm with an SUVmax of 4, while the most prominent bone lesion showed an SUVmax of 5.2 (**Figures 4A–C**).

**Figure 4:**
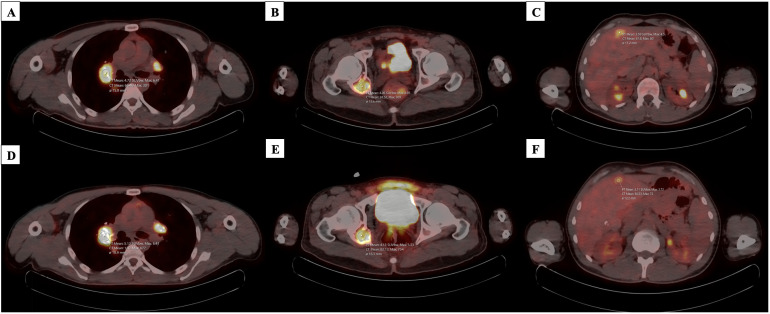
Comparison of baseline (2024) and follow-up (2025) PET/CT images. **(A–C)** Baseline PET/CT demonstrating FDG-avid lesions in the right hilum, ischium, and liver, respectively. **(D–F)** Follow-up PET/CT in March 2025 showing persistent lesions with stable size and mild variations in metabolic activity (SUVmax) in the same organs, respectively, consistent with stable disease.

On follow-up, PET/CT in March 2025, these lesions remained stable in size. The right hilar lesion measured 4.1 cm, with a slight increase in SUVmax to 8.4. The hepatic lesion remained stable at 2.4 cm with a slight decrease in SUVmax to 3.7, while the bone lesion showed a mild increase in SUVmax to 6.1 (**Figures 4D–F**).

According to these PET/CT findings, the lesion sizes remain stable despite minor variations in metabolic activity.

He maintained radiographic stability for over 4 years, with serial imaging (including CT and PET/CT). According to the Response Evaluation Criteria in Solid Tumors (RECIST) version 1.1, imaging of measurable main lesions in the hilum, ischium and liver does not demonstrate sufficient tumor shrinkage to qualify as a partial response (PR) (≥30% decrease in lesion size) or a sufficient increase to qualify as progressive disease (PD) (≥20% increase or development of new lesions). Therefore, our patient was classified as having stable disease (SD).

Throughout the course of treatment, the dose of Pazopanib has not changed (remained 800 mg once daily), although the patient developed characteristic side effects of TKI therapy, most notably poliosis. In September 2025, his clinical course was complicated by the new onset of significant gastrointestinal toxicity, predominantly severe postprandial diarrhea, with loose stools after each meal (more than 5 times daily) but without obvious blood or any discoloration, which affected his daily activities and required 2 admissions to a local hospital for dehydration management. Therefore, it was considered grade 3 diarrhea according to the Common Terminology Criteria for Adverse Events (CTCAE). Due to that, a prolonged treatment interruption (“drug rest”) was initiated, and diarrhea started to resolve only after one month of drug discontinuation.

As of early 2026, the patient remains in this treatment-free interval. Remarkably, despite cessation of Pazopanib in September 2025, serial imaging continues to show sustained radiographic stability. Since his diagnosis at our hospital, his Eastern Cooperative Oncology Group (ECOG) performance score has remained 0, as he has been fully active and able to carry out all pre-disease activities without restriction. This longitudinal course, now approaching five years since diagnosis, demonstrates both the efficacy of Pazopanib in achieving long-term control and the potential for a stable, indolent disease state even during treatment-free intervals.

## 3 Discussion

### 3.1 Overview of EHE and HEHE

Cutaneous and soft-tissue EHEs tend to follow an indolent course, while visceral lesions, particularly hepatic and pulmonary, are associated with higher rates of multifocality and metastasis. At the molecular level, 90% of EHE variants express a WWTR1-CAMTA1 fusion, which activates the Hippo pathway and drives endothelial proliferation. It typically presents with an indolent-to-moderately aggressive course, characterized by epithelioid endothelial cells in a fibromyxoid stroma. A 10% express YAP1-TFE3 gene fusion was associated with a younger age group (30s), more aggressive spread, and shorter survival (11). Although no molecular or genetic testing has been conducted in the case, the patient’s young age and extensive multiorgan involvement, even with low Ki-67, may suggest that he has another driver leading to this aggressiveness beyond the proliferative index alone, which can be the YAP1-TFE3 gene fusion molecular pathway. In addition, tumor cells can produce angiogenic mediators such as VEGF and monocyte chemoattractant protein 1, which promote endothelial proliferation, and this may also explain the tumor’s tendency to hematogenous spread to the liver, lungs, pleura, bone, and lymph nodes (12, 13).

A notable variant is HEHE, which refers to cases in which this vascular tumor is predominantly found in the liver parenchyma; it was first described by Ishak in 1984 (14). It originates from endothelial cells lining hepatic sinusoids and small veins and is the most commonly documented subtype. The mean age at diagnosis is 41.7 years, and it is more commonly seen in females (2:3 ratio). The median overall survival in HEHE is 16.9 years. The onset of HEHE is typically insidious, and most cases are diagnosed at the middle or late stage (15).

### 3.2 Clinical presentation

The common presenting patients’ complaints of HEHE are epigastric pain, a poor appetite and fatigue. Fever and jaundice are occasionally observed. Metastasis occurs in one-third of HEHE cases due to the liver’s rich blood supply. It may also migrate through the portal vein to the lungs or the abdomen, which then causes liver and respiratory failure. HEHE can present as a polycentric origin tumor with multiple primary lesions at the same time, which can be difficult to distinguish from metastasis (16).

### 3.3 Imaging findings

It is most commonly detected incidentally on imaging, with multiple lesions observed beneath the liver capsule or around the liver. On ultrasonography, the most common finding is hypoechoic masses, although heterogeneous or hyperechoic masses may also be observed, depending on the tumor stage (17). On non-contrast CT, the tumor nodules most commonly have a hypodense circular appearance, and when the enhancing contrast is administered, small nodules predominantly have a homogeneous look, while larger nodules show a heterogeneous appearance with peripheral enhancement, giving some characteristic features like the ‘lollipop’ sign; occurring when a tapered occluded vein ends abruptly on the margin of the mass, capsular retraction, calcification, halo sign, and target sign (18). In our case, the presence of the lollipop sign strengthened suspicion for the diagnosis and helped distinguish it from other differentials, such as HCC and angiosarcoma, given the chaotic vascular destruction in both. Magnetic resonance imaging demonstrates a well-defined tumor with hypointensity on T1-weighted images and hyperintensity on T2-weighted images. Larger lesions (>2 cm) are prone to liquefaction necrosis, and the lesion density or signal is uneven (19).

### 3.4 Histopathology and IHC

HEHE is misdiagnosed in 60% to 80% of cases due to its exceptionally low prevalence, the limited diagnostic value of physical examination, and the aforementioned indistinguishable radiological features, which appear similar to other hepatic malignancies such as metastatic carcinoma, angiosarcoma, the sclerosing variant of hepatocellular carcinoma, and cholangiocarcinoma (17). Therefore, the definitive diagnosis of HEHE cannot be confirmed without a biopsy. Microscopically, the tumor cells exhibit a dense, disorganized architecture with cord-like or nested cord-like formations. The cells display epithelioid, spindle-shaped, or irregular morphology. Their nuclei are enlarged and irregular, with chromatin distributed unevenly or coarsely granular (20). In immunohistochemistry (IHC), vascular structures are identified by positive staining for endothelial markers such as Factor VIII-related antigen, CD34, CD31, ERG, and FLI-1. Other IHC stains that can be positive include factor XIIIa, vimentin, and type IV collagen (5).

### 3.5 Differential diagnosis

HEHE should be differentiated from other primary and secondary liver tumors, especially HCC and angiosarcoma. HCC, the most common primary liver tumor, demonstrates characteristic imaging findings such as arterial-phase hyperenhancement and delayed-phase washout. In addition, the alpha-fetoprotein serum marker is elevated in HCC, with a previous history of underlying chronic liver pathology in most cases. On the contrary, HEHE has multifocal lesions and characteristic imaging findings such as the lollipop and target signs (21).

Angiosarcoma, which is also a vascular tumor that can arise in the liver, tends to have a rapid progression course with extensive hemorrhage. Although both tumors can express endothelial markers, the presence of higher mitotic activity and marked cellular atypia is more characteristic of angiosarcoma. In addition, radiological findings (e.g., lollipop sign) are more consistent with HEHE than with angiosarcoma (21, 22).

In our case, the characteristic imaging and histopathological findings support the diagnosis of HEHE rather than other liver malignancies.

### 3.6 Management and treatment

Once the diagnosis is confirmed, a multidisciplinary tumor board is essential to determine the optimal treatment for each patient. Therapeutic experience has remained anecdotal: The management options for hepatic EHE include liver resection, liver transplantation, chemotherapy, radiotherapy, immunotherapy, or surveillance alone (23). Antiangiogenic agents have produced partial responses or disease stabilization in small prospective and retrospective studies, and immune checkpoint inhibitors are also being investigated in early-phase studies in this population (24, 25).

For asymptomatic patients, the initial recommended option is active surveillance. Regarding unifocal EHE, the treatment of choice is surgery, aiming for complete resection of the tumor with microscopic negative (R0) margins. The expected cure rate for these patients is 70%-80%. If there are positive margins (R1), the surgery can be supplemented with radiation therapy. If the patient has severe comorbidities that make them ineligible for complete resection (R0 or R1), surgery can be replaced with definitive radiotherapy, radiofrequency ablation, or microwave ablation; the best alternative is determined by several factors, including the lesion’s size and anatomical location. It is important to note that there is no role for neoadjuvant therapy for a resectable unifocal tumor (7).

As for multifocal hepatic EHE, it can remain stable for many years without active treatment, but data on its management remain limited. LT should be offered as a treatment modality for patients with unresectable disease. It is also an option for patients with EHE confined to the liver who develop liver failure due to the tumor, but are generally in good condition. The overall survival rate after the transplant, according to a study of 149 patients, is 7.6 years. The history of tumor rupture should be considered a major contraindication to LT because it’s a risk factor for recurrence (7).

Several chemotherapy regimens have been suggested in metastatic hepatic EHE, yet no standard treatment has been established. Historically used therapies include doxorubicin, vincristine, interferon-alpha, 5-FU, thalidomide, and anti-VEGF agents such as monoclonal antibodies (bevacizumab, ranibizumab), aptamers (pegaptanib), and VEGF receptor antagonists (sunitinib) (26). Case histories show variable outcomes: one patient, treated with doxorubicin, vincristine, and 5-FU, had no symptoms for 10 years; another, with spontaneous remission after doxorubicin; and a third, refractory to epirubicin and dacarbazine. The anti-angiogenic effects of thalidomide have been extremely effective.

Pazopanib has recently received approval from both the U.S. Food and Drug Administration (FDA) and the European Medicines Agency (EMA) for the treatment of advanced renal cell carcinoma and soft tissue sarcomas (STS) (27). It is a tyrosine kinase inhibitor (TKI) with high affinity for VEGFR-1/2/3 and lower affinity for PDGFR-α/β, FGFR-1/2, and stem cell factor receptor (c-KitR). VEGFR and FGFR play an important role in endothelial proliferation and angiogenesis. Hence, when Pazopanib blocks these receptors, it inhibits angiogenesis in endothelial vascular tumors that express high levels of these receptors, such as EHE. In addition, PDGFR plays a synergistic role by supporting stromal cells and the tumor microenvironment, which adds benefits and more efficacy to the drug by suppressing it. This mechanism of action may contribute to the suppression of tumor proliferation and lead to stabilization (28).

Pazopanib has shown promising results in metastatic EHE that cannot be surgically removed, or as an alternative to mTOR inhibitors (e.g., Sirolimus), with prolonged stabilization associated with significant intratumoral changes (28).

In a retrospective study of 13 patients with metastatic EHE, first-line Pazopanib showed clinical activity, similar to sirolimus, with a median progression-free survival of 35.1 months and a median overall survival of 53.8 months. Although no complete or partial responses were observed, a clinical benefit was achieved in 62% of patients, with some experiencing symptom improvement. Toxicity was generally manageable, primarily gastrointestinal, with few treatment discontinuations. The study supports Pazopanib as a safe and active first-line option in metastatic EHE (29).

In a reported case, a female patient with HEHE was treated with Pazopanib and achieved long-term disease control. The patient received continuous Pazopanib therapy for nearly eight years, during which she experienced clinical improvement of symptoms and sustained radiological stability of both hepatic tumors and pulmonary lesions (28).

Given the lack of approved therapies for EHE and Pazopanib’s mechanism of action, regulatory approval, and established use in soft tissue sarcomas (STS), Pazopanib was adopted as a first-line treatment for our patient.

Pazopanib is generally well tolerated with few side effects. Common side effects (≥10–20% of patients) include gastrointestinal disturbances (such as diarrhea, nausea, vomiting, abdominal pain, or decreased appetite), hair color changes (lightening of hair), skin hypopigmentation, hypertension, palpitations, and hand-foot skin reaction (HFSR) (30). The latter manifests as tender palmoplantar lesions, especially in areas of trauma or friction; this is considered a cause for discontinuation of the drug (31). Cyclophosphamide metronomic chemotherapy, administered continuously at low doses, has also demonstrated immunologic and anti-angiogenic activity (15).

Data regarding disease behavior after discontinuation of Pazopanib in EHE are limited, and most published reports describe disease control during continuous therapy rather than after treatment cessation.

In Palestine, the management of cancer is significantly affected by structural, financial, and logistical constraints within the healthcare system. Limited public healthcare funding and restricted oncology budgets contribute to inconsistent availability of advanced anticancer therapies, particularly novel targeted and high-cost agents. Consequently, access to many cancer treatments often depends on out-of-pocket payments, private healthcare coverage, and external funding mechanisms. In addition, administrative and logistical barriers, including prolonged referral and transfer processes between healthcare facilities, may delay the timely initiation of therapy. Requests for advanced treatments are also subject to approval processes that may result in frequent denials or partial coverage, further limiting access to treatment. These challenges can collectively lead to delays in optimal care, interruptions in treatment continuity, and reliance on adapted regimens in the absence of standardized disease-specific protocols, particularly in rare malignancies.

If a patient with EHE received no treatment, it was found that survival rates decreased dramatically, with a 1-year survival of 39.3% and a 5-year survival of only 4.5% and according to a study of 228 patient cases with hepatic epithelioid hemangioendothelioma, researchers determined that multifocality, lesion size larger than 10 cm, and extrahepatic disease reduced five-year survival to half that of localized disease (31).

## 4 Patient perspective

The patient reported that receiving the diagnosis had a significant psychological impact on him. It was shocking, particularly due to the prolonged period required to reach a definitive diagnosis. During that time, he was unable to continue working, which further contributed to his emotional distress. However, after establishing the diagnosis and initiating treatment with Pazopanib, his symptoms improved, allowing him to gradually return to work.

He described himself as a patient and resilient individual, emphasizing the strong support he received from his family throughout his illness. He also reported a positive experience with the medical team and expressed satisfaction with the care provided.

Nevertheless, he highlighted certain challenges in accessing healthcare, particularly difficulties obtaining permits to reach the hospital and receive treatment. As a result, he occasionally had to obtain the medication from local sources at a higher cost.

## 5 Conclusion

In our case, HEHE presented with an atypical presentation, a prominent intracranial mass, and extensive metastatic disease at the time of diagnosis, which made surgical treatment difficult. Therefore, a biological therapy with Pazopanib was initiated, based on prior literature recommending starting it when the tumor is surgically unresectable, multifocal, or when prominent metastatic symptoms are present, such as the progressive headache in our patient. Due to unclear available management guidelines of disseminated EHE, the treatment plan included continuing the drug unless new lesions or unanticipated side effects (grade 3 or 4) occurred. The patient underwent serial follow-up, including physical examination and imaging, which revealed stable disease. He reports no change in functional status. This case demonstrates the potential effectiveness of Pazopanib, which we recommend physicians consider as a therapeutic option, especially for patients with multifocal presentation. One limitation of this paper is the lack of molecular or genetic testing, and the study is a case report due to the disease’s rarity. Accordingly, we suggest that future papers use a prospective study design involving multiple patients to detect favorable clinical outcomes and investigate the mechanisms underlying long-term stability even after drug withdrawal.

## Data Availability

The original contributions presented in the study are included in the article/supplementary material. Further inquiries can be directed to the corresponding author.
